# Data on physicochemical quality of drinking water in the rural area in Divandarreh county, Kurdistan, Iran

**DOI:** 10.1016/j.dib.2018.06.058

**Published:** 2018-06-22

**Authors:** Afshin Maleki, Bahram kamarehie, Reza Rezaee, Ali Jafari, Yahya Zandsalami, Pegah Bahmani, Esmail Ghahramani, Mohammad Amin Karami

**Affiliations:** a Environmental Health Research Center, Research Institute for Health Department, Kurdistan University of Medical Sciences, Sanandaj, Iran; b Department of Environmental Health Engineering, Faculty of Health and Nutrition, Lorestan University of Medical Sciences, Khorramabad, Iran; c Environmental Health Research Center, Kurdistan University of Medical Sciences, Sanandaj, Iran

**Keywords:** Divandarreh, Physicochemical quality, Drinking water, Rural area

## Abstract

Good quality of drinking water is very important in the maintenance of human health. The gathered data from the present work was used to evaluate the quality of drinking water resources in the rural villages of Divandarreh, Iran. Physicochemical quality of water was determined by a collection of 35 random samples during dry and rainy seasons in 2015. The APHA approach was used to determine the physicochemical parameters of the samples. The results showed that the average concentration of Ca, Mg, Na, K, Cl, SO_4_, TDS and TH during dry season was 85.64 mg/l, 13.41 mg/l, 34.11 mg/l, 2.8 mg/l, 9.9 mg/l, 45.7 mg/l, 326.06 mg/l and 269.61 mg/l, respectively. Also, the average concentration of the parameters during rainy season was 77.3 mg/l, 18.27 mg/l, 30.3 mg/l, 1.9 mg/l, 12.54 mg/l, 39 mg/l, 269.1 mg/l and 316.17 mg/l, respectively.

**Specifications Table**

**Table:** 

Subject area	Chemistry
More specific subject area	Drinking water quality
Type of data	Tables and map
How data was acquired	Analyses of temporary hardness, calcium, magnesium were conducted using titration method. The electrical conductivity and pH of samples were determined with (Jenway 470 Conductivity meter) and pH meter (model Jenway 350), respectively. Measurement of sulfate anions and cations was done by spectrophotometry (DR 5000; Hach) in water.
Data format	Analyzed
Experimental factors	The water samples stored at room temperature and were analyzed according to the APHA approach.
Experimental features	The levels of physical and chemical parameters were determined.
Data source location	Divandarreh, Kurdistan province, Iran
Data accessibility	Data are included in this article

**Value of the data**

• Based on the obtained data proper measure can be taken in order to deliver appropriate water quality to consumers.
• The collected data can be used for the preparation of research map in the field of water treatment by another researcher.
• The collected data can be useful for the codification of local standards along with other researcher׳s data.

## 1 Data

**Fig. 1:**
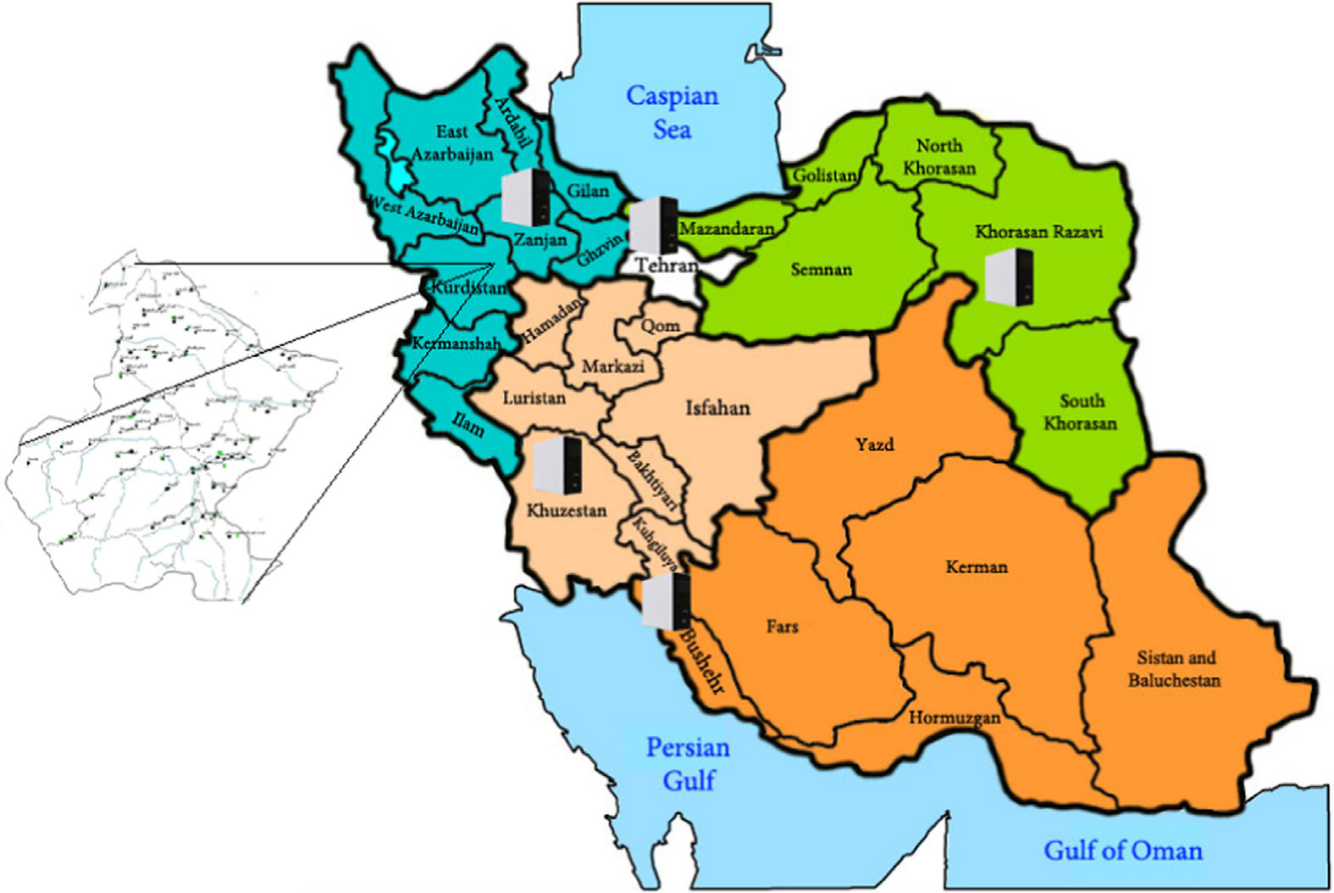
Location and sampling points in Divandarreh city.

The specified parameters in the experiments are included calcium ion, magnesium ion, sodium ion, chloride ion, sulfate ion, temperature, total alkalinity, electrical conductivity (EC), total dissolved solids (TDS) and pH analyzed according to standard methods for the examination of water and wastewater [1]. Water stability was evaluated based on RSI, LSI, PSI, AI and LS. The sampling points and study area was shown in Fig. 1. The chemical and physical characteristics of intended water samples are presented in Tables 1 and 2. Tables 3 and 4 shows the equations and indicators for categorizing the water stability. Calculation of water quality indices is shown in Table 5. Table 6 illustrates the stability indices in water resource. The condition of water stability in the water resources is shown in Table 7.

**Table 1:** Analyses of water quality parameters of the study area during dry seasons in 2015.

**Sample nos.**	**Ca**^**+2**^**(mg/l)**	**Mg**^**+2**^**(mg/l)**	**Na**^**+**^**(mg/l)**	**K**^**+**^**(mg/l)**	**Cl^−^ (mg/l)**	${SO}_{4}^{-2}$**(mg/l)**	**Alk**	**TH (mg/l)**	**pH**	**EC (μmhos/cm)**	**TDS (mg/l)**	**Tem C**
1	64	19	15	2.4	5.58	7.05	276	238.9	7.88	437	283	22.1
2	64	9.7	9	0.4	2.90	10.7	200	200.6	7.7	323	209	21.2
3	96	25	31	1.8	13.18	45.6	380	343.8	7.64	642	417	20.3
4	128	20	71	4.4	12.55	57.7	460	403	7.75	847	550	21.7
5	72	10	26	1	8.49	38.8	254	221.5	8.04	419	273	21.4
6	64	5	14	0.4	2.93	16.6	150	180.8	7.93	267	173	19.7
7	64	10.2	19	0.4	2.36	26.2	264	202.33	7.34	406	264	20.5
8	62	10	10	0.7	2.54	12.9	140	196.5	7.57	245	159	21.2
9	48	15	8	0.3	1.15	7.8	140	182.25	7.62	198	128	21.8
10	48	8	8	0.6	3.07	14	190	153.2	7.77	281	182	20.3
11	51.2	9	8	0.4	1.56	9.6	120	165.4	7.84	190	122	20.8
12	43.2	20.5	6	0.3	2.09	19.9	230	193.06	7.82	351	228	19.9
13	63.2	14.5	10	0.4	2.48	46.3	210	218.18	7.56	410	268	19.2
14	82.8	13	3	0.3	1.57	32.6	220	261	8.1	299	194	19.7
15	58.4	10.5	11	0.2	1.744	17.5	190	189.58	7.69	300	195	19.8
16	59.2	3	10	0.3	12.02	25.2	210	160.5	7.81	395	256	19.8
17	72	12.7	14	2.9	3.96	21.7	240	232.7	7.88	407	266	20.3
18	76	2.6	37	0.5	25.69	65.4	340	200.8	7.73	574	373	19.3
19	111.2	3.1	46	4.2	27.715	62.2	384	290.87	6.93	630	411	20
20	104	4.5	75	5	13.33	71.7	396	278.68	8.12	862	560	20.5
21	112	3.7	56	0.1	19.89	59.8	260	295.4	7.46	489	317	19.8
22	55.2	5.4	89	13.6	24.39	54.9	420	160.41	7.37	836	545	19.8
23	92	2.5	76	6.3	15.082	60.6	380	240.4	7.61	651	421	20.4
24	72	3.5	94	11.8	13.72	60.2	480	194.53	7.44	1141	741	20.2
25	211	2.5	75	6.2	19.3	70.3	380	537.88	7.95	928	605	20
26	123	2	58	2.8	20.47	76.5	340	315.8	7.1	563	366	20.5
27	95.2	1.6	72	2.4	15.27	63.1	340	244.64	8.15	694	450	19.7
28	98.4	6	67	3	11.63	42.8	230	270.9	7.88	532	345	18.8
29	71.2	1	90	8.4	19.781	0	450	182.15	7.58	1089	708	18.9
30	120	30	14	1.6	12.51	44.9	120	424.5	7.69	193	125	18.7
31	40	30	18	7.5	3.33	0	220	224.5	7.83	367	239	19
32	68	44	9	1	2.70	364	160	352.6	7.6	281	182	19.6
33	60	30	13	4.6	4.03	24.3	230	274.5	7.73	330	213	18.8
34	156	40	16	1	5.56	33.2	210	556	8.04	396	258	20
35	190	42	16	0.5	11.99	34.8	260	649.3	8.28	434	281	20.2
**Max**	211	44	94	13.6	27.71	364	480	649.3	8.28	1141	741	22.1
**Min**	40	1	3	0.1	1.15	0	120	153.2	6.93	190	122	187
**SD**	39.5	12.3	30	3.4	8	59.84	103	118.23	0.29	251.6	163.8	0.87
**Mean**	85.64	13.41	34.11	2.8	9.9	45.7	270.7	269.61	7.73	497.35	323.06	20.11

**Table 2:** Analyses of water quality parameters of the study area during rainy seasons in 2015.

**Sample No**	**Ca^+2^ (mg/l)**	**Mg^+2^ (mg/l)**	**Na^+^ (mg/l)**	**K^+^ (mg/l)**	**Cl^−^ (mg/l)**	${SO}_{4}^{-2}$**(mg/l)**	**Alk**	**TH (mg/l)**	**pH**	**EC (μmhos/cm)**	**TDS (mg/l)**	**Tem C**
1	93.3	26	29	0.2	12.8	26.57	400	341.15	7.41	577	375	22
2	65.6	18.5	6	0.3	4.19	15.8	180	240.78	7.21	317	205	22
3	112	22	27	1.1	15.6	48.4	340	371.3	7.32	605	393	21.5
4	60	17.07	16	1.3	6.54	36.1	180	220.84	7.11	301	196	21.8
5	64	19.5	14	0.2	3.73	31.9	200	240.93	7.3	386	251	21.8
6	51.5	12.5	14	0.2	3.83	16.6	140	180.63	7.14	274	180	21.9
7	66	13.3	13	0.2	1.65	29.8	200	220.2	6.95	336	219	21.8
8	48	9.7	7	0.3	1.84	17.3	140	160.3	7.34	220	143	21.6
9	93	16.6	20	3.8	9.83	21	260	301.39	7.32	535	346	21.7
10	54	11	4	0.3	2.61	21.3	160	180.65	7.11	282	183	21
11	68	14.6	12	1.1	3.27	23.6	240	230.59	7	396	257	21.8
12	59	12.7	4	0.2	2.60	24.2	140	200.20	6.7	302	197	21.2
13	72	22	6	0.3	2.91	42.9	180	271.3	7.3	428	277	21.8
14	66.3	13.2	2	0.2	1.57	32.6	190	220.53	7.1	315	205	21.6
15	57.1	11.5	8	0.2	2.40	34.2	170	190.48	7.25	347	225	21.7
16	48	9.7	7	0.2	3.67	19.4	90	160.26	7.2	175	114	21.6
17	56	9.7	7	0.2	1.5	53.2	150	180.26	7.34	271	177	21.2
18	92	19.5	55	3.3	36.84	38.9	280	310.93	7.69	651	423	21.9
19	56	22	20	3.1	19.35	45.4	210	231.3	7.33	377	246	21.9
20	96	24.3	56	12.1	23.4	52.2	260	340.84	7.8	659	428	22.2
21	85.4	18.6	52	5.4	16.4	40.8	220	290.69	7.3	491	316	22.8
22	99	27.4	85	14.9	35.08	58.4	380	361.21	7.69	853	557	22.9
23	84	17	72	4.2	19.18	57.5	320	280.55	7.7	644	419	22.2
24	152	38.7	84	2	15.82	50.2	360	540.60	7.85	1103	775	21.2
25	112	29.3	66	1.2	28	59.4	310	401.6	7.74	884	575	21.5
26	90	22.6	52	1.3	21.6	85.1	300	318.79	7.41	586	380	21.6
27	81	21.3	94	5.4	20.6	25.9	260	290.9	7.6	701	454	21.4
28	69	18.8	64	0.4	19.6	75.7	210	250.52	7.1	534	347	21.6
29	155	37.3	96	1.4	33.86	43.1	420	542.3	7.9	1152	749	21.6
30	50	8.9	7	0.2	8.5	50.5	100	161.94	7.19	210	137	22
31	71.1	17.6	16	0.2	21.74	45.3	230	250.79	7.3	470	306	22.2
32	51	8.2	6	0.3	4	36.2	170	161.53	6.9	318	206	21.4
33	77.2	16.3	11	0.2	12	35.1	160	260.65	6.95	390	254	22.3
34	75.3	17.5	14	0.3	8.019	42.3	180	260.87	7.6	420	273	20.9
35	76	14.6	14	0.2	14.4	26.5	210	250.59	7.1	426	278	20.9
**MAX**	155	38.7	96	14.9	36.84	85.1	420	542.3	7.9	1152	775	22.9
**MIN**	48	8.2	2	0.2	1.5	15.8	90	160.25	6.7	175	114	20.9
**SD**	26	7.3	29.67	3.29	10.47	16.41	84	93.22	0.3	236.8	158.6	0.45
**Mean**	77.3	18.27	30.3	1.9	12.54	39	226.9	269.1	7.3	483.9	316.17	21.73

**Table 3:** List of indices and water quality in present work.

Water quality parameter	Formula	References
Soluble Sodium Percentage (SSP)	SSP=$\frac{\mathrm{Na}+K}{\mathrm{Ca}+\mathrm{Mg}+\mathrm{Na}+K}*100$	[8]
Kelly׳s Ratio(KR)	KR=$\frac{\mathrm{Na}}{\mathrm{Ca}+\mathrm{Mg}}$	[7]
Permeability Index (PI)	PI =$\frac{\mathrm{Na}+K+\surd {\mathrm{HCO}}_{3}}{\mathrm{Ca}+\mathrm{Mg}+\mathrm{Na}+K}*100$	[7]
Sodium Adsorption Ratio (SAR)	SAR=$\frac{\mathrm{Na}}{\surd (\frac{\mathrm{Ca}+\mathrm{Mg}}{2})}*100$	[5], [7]
Magnesium Hazard (MH)	MH=$\frac{\mathrm{Mg}}{\mathrm{Ca}+\mathrm{Mg}}$	[7]

**Table 4:** Corrosion and saturation indicator, equation and criteria for categorizing the stability of the water used in the present work [4], [5].

**Index**	**Equation**	**Index value**	**Water condition**
Ryznar stability index (RSI)	RSI=2pHs–pH	RSI<6	Super saturated
	pHs=A+B–log (Ca^2+^)–log (Alk)	6<RSI<7	Saturated
		RSI>7	Under saturated
Langelier saturation index (LSI	LSI=pH–pHs	LSI>0	Super saturated
		LSI=0	Saturated
		LSI<0	Under saturated
Aggressive index (AI)	AI=pH+log[(alk)(H)]	AI>12	Non aggressive
		10<AI<12	Moderately aggressive
		AI<10	Very aggressive
Puckorius scaling index (PSI)	PSI=2 (pHeq)–pHs	PSI<6	Scaling is unlikely to occur
	pH=1.465+log	PSI>7	Likely to dissolve scale
	(T.ALK)+4.54		
	pHeq 1.465×log		
	(T.ALK)+4.54

**Table 5:** Calculation of water quality indices in present work.

**Sample**	**Dry season**					**Rainy season**				
	**SSP**	**KR**	**PI**	**MH**	**SAR**	**SSP**	**KR**	**PI**	**MH**	**SAR**
1	13.03	0.14	51.86	32.9	0.422	15.69	0.18	47.41	31.5	0.68
2	9.13	0.1	50.28	20	0.28	5.3	0.05	39.17	31.76	0.16
3	16.9	0.2	47.14	30	0.73	13.96	0.16	41.37	24.5	0.60
4	28.45	0.38	52.87	20.5	1.54	14.2	0.158	47.66	32	0.47
5	20.72	0.26	57.3	18.7	0.76	11.33	0.127	44.74	33.46	0.4
6	14.63	0.17	51.7	11.42	0.45	14.55	0.17	50.47	28.6	0.45
7	17.15	0.2	59.82	20.82	0.58	11.49	0.13	47.96	25	0.38
8	10.34	0.11	45	21	0.312	8.9	0.098	52.04	25	0.24
9	8.91	0.096	46.9	34	0.26	13.85	0.14	43.41	22.75	0.5
10	10.61	0.114	62.19	21.6	0.28	4.8	0.05	47.56	25.15	0.13
11	9.79	0.10	48.12	22.5	0.27	10.68	0.11	49.18	26.1616	0.34
12	6.52	0.068	53.7	43.9	0.18	4.29	0.04	40.58	26.21	0.12
13	9.27	0.1	47.94	27.5	0.3	4.73	0.04	34.98	33.53	0.16
14	2.58	0.06	38.09	20.56	0.08	2.05	0.02	41.33	24.73	0.06
15	11.39	0.127	52.68	22.9	0.35	8.5	0.09	48.7	24.94	0.25
16	12.12	0.136	62.97	7.7	0.34	8.82	0.09	43.5	25	0.24
17	12.81	0.13	50.05	22.5	0.4	7.92	0.085	48.05	22.23	0.23
18	28.77	0.4	70.67	5.3	1.13	28.52	0.39	53.20	25.91	1.35
19	26.60	0.344	58.27	4.4	1.173	17.07	0.19	50.44	39.33	0.57
20	37.83	0.58	66.27	6.7	1.95	28.76	0.36	50.39	29.5	1.32
21	29.21	0.41	53.97	5.2	1.417	29.26	0.39	52.42	26.44	1.32
22	56.83	1.2	92.18	13.9	3.05	36.14	0.513	58.26	31.35	1.94
23	41.90	0.7	72.07	4.3	2.13	36.64	0.56	62.56	25.03	1.87
24	53.03	1.05	86.91	7.4	2.93	25.56	0.34	42.33	29.6	1.57
25	24.12	0.30	41.73	1.9	1.40	26.58	0.36	47.24	30.15	1.43
26	29.11	0.4	55.61	2.6	1.42	26.51	0.36	52.13	29.3	1.27
27	39.49	0.64	68.69	2.7	2.0	42.13	0.70	62.72	30.26	2.4
28	35.58	0.54	58.68	9	1.77	35.85	0.56	59.67	31.01	1.76
29	53.18	1.07	88.08	2.3	2.9	28.01	0.39	45.47	28.42	1.8
30	7.12	0.07	22.50	29	0.3	8.74	0.09	44.89	22.7	0.24
31	17.90	0.17	52.79	55	0.52	12.28	0.14	46.33	29	0.44
32	5.60	0.056	27.38	51.64	0.20	7.69	0.08	55.47	21	0.2
33	11.10	0.10	42.67	45	0.34	8.50	0.09	37	25.84	0.3
34	6.10	0.063	21.81	29.7	0.3	10.59	0.11	40.1	27.7	0.38
35	5.18	0.054	20.3	26.7	0.27	10.93	0.12	44	24.07	0.38
**Max**	56.83	1.2	92.18	55	3.05	42.13	0.7	62.72	39.33	2.4
**Min**	2.58	0.02	20.3	1.9	0.08	2.05	0.02	35	21	0.06
**SD**	14.96	0.30	16.80	14.7	0.87	10.99	0.17	6.69	3.9	0.66
**Aver**	20.66	0.303	53.69	20	0.94	16.6	0.21	47.8	27.7	0.74

**Table 6:** Calculation stability indexes in water resource in the present work.

**Sample**	**Dry season**				**Rainy season**			
	**RSI**	**LSI**	**AI**	**PSI**	**RSI**	**LSI**	**AI**	**PSI**
1	6.72	0.59	12.52	7.8	6.6	0.41	12.4	8.10
2	6.9	0.27	12.2	7.53	7.7	−0.23	11.7	7.44
3	7.21	0.59	12.6	8.06	6.7	0.31	12.3	8
4	6.4	0.90	12.92	8.22	7.86	−0.37	11.54	7.44
5	5.65	0.75	12.7	7.73	7.55	−0.12	11.81	7.52
6	6.7	0.36	12.31	7.3	8.16	−0.515	11.4	7.23
7	7.80	−0.002	12	7.8	7.9	−0.46	11.47	7.52
8	7.11	−0.008	11.91	7.23	8	−0.33	11.6	7.22
9	7.54	−0.042	11.84	7.23	7.05	0.137	12.10	7.75
10	7.56	0.184	12.13	7.482	8.08	−0.48	11.45	7.34
11	7.33	0.119	12.03	7.1	7.70	−0.38	11.56	7.7
12	7.62	0.246	12.215	7.64	8.53	−0.914	11.01	7.23
13	7.6	0.084	12.08	7.6	7.6	−0.13	11.81	7.44
14	6.9	0.8	12.8	7.605	7.8	−0.33	11.6	7.5
15	7	0.173	12.13	7.5	7.84	−0.3	11.64	7.4
16	7.22	0.32	12.30	7.6	8.50	−0.7	11.23	6.9
17	7.1	0.54	12.51	7.7	7.9	−0.26	11.7	7.3
18	7	0.51	12.54	8	6.7	0.52	12.5	7.80
19	7.50	−0.06	12	8.07	7.6	−0.13	11.8	7.6
20	5.9	1.09	13.13	8.1	6.6	0.62	12.6	7.75
21	6.6	0.323	12.32	7.75	7.22	0.04	11.97	7.60
22	7	0.08	12.13	8.15	6.34	0.68	12.66	8.062
23	6.973	0.54	12.55	8.063	6.6	0.56	12.53	7.92
24	6.70	0.3	12.4	8.26	6.01	0.92	13	8.0
25	6.34	1.19	13.25	8.063	6.43	0.66	12.7	7.86
26	6.42	0.12	12.12	8	6.9	0.27	12.24	7.87
27	5.80	1.020	13.06	8	7	0.32	12.32	7.74
28	6.38	0.60	12.63	7.64	7.71	−0.30	11.7	7.6
29	7	0.38	12.5	8.2	5.8	1.06	13.11	8.15
30	6.71	0.29	12.24	7.1	8.4	−0.60	11.3	6.94
31	6.98	0.18	12.17	7.6	7.37	−0.03	11.91	7.64
32	7.70	0.075	12.0	7.34	8.3	−0.7	11.24	7.39
33	7.32	0.28	12.27	7.64	7.92	−0.49	11.44	7.34
34	6.9	0.98	13	7.6	7.3	0.17	12.13	7.44
35	5.85	1.4	13.4	7.74	7.62	−0.26	11.7	7.57
**Max**	7.80	1.4	13.4	8.26	8.5	1.06	13.1	8.15
**Min**	5.65	−0.06	11.84	7.1	5.8	−0.914	11.01	6.86
**SD**	0.56	0.38	0.4	0.33	0.72	0.5	0.53	0.31
**Aver**	6.9	0.43	12.42	7.71	7.4	−0.03	12	7.6

**Table 7:** Water stability conditions in present work.

**Sample**	**Dry season**				**Rainy season**			
	**RSI**	**LSI**	**AI**	**PSI**	**RSI**	**LSI**	**AI**	**PSI**
1	S^*^	St	NA^*^	Ct	S	St	NA	Ct
2	S	St	NA	Ct	Ct	Ct	MA	Ct
3	Ct^**^	St	NA	Ct	S	St	NA	Ct
4	S	St	NA	Ct	Ct	Ct	MA	Ct
5	St^***^	St	NA	Ct	Ct	Ct	MA	Ct
6	S	St	NA	Ct	Ct	Ct	MA	Ct
7	Ct	Ct	MA^**^	Ct	Ct	Ct	MA	Ct
8	Ct	Ct	MA	Ct	Ct	Ct	MA	Ct
9	Ct	Ct	MA	Ct	Ct	St	NA	Ct
10	Ct	St	NA	Ct	Ct	Ct	MA	Ct
11	Ct	St	NA	Ct	Ct	Ct	MA	Ct
12	Ct	St	NA	Ct	Ct	Ct	MA	Ct
13	Ct	St	NA	Ct	Ct	Ct	MA	Ct
14	S	St	NA	Ct	Ct	Ct	MA	Ct
15	S	St	NA	Ct	Ct	Ct	MA	Ct
16	Ct	St	NA	Ct	Ct	Ct	MA	Ct
17	Ct	St	NA	Ct	Ct	Ct	MA	Ct
18	S	St	NA	Ct	S	St	NA	Ct
19	Ct	Ct	MA	Ct	Ct	Ct	MA	Ct
20	St	St	NA	Ct	S	St	NA	Ct
21	S	St	NA	Ct	Ct	St	MA	Ct
22	S	St	NA	Ct	S	St	NA	Ct
23	S	St	NA	Ct	S	St	NA	Ct
24	S	St	NA	Ct	S	St	NA	Ct
25	S	St	NA	Ct	S	St	NA	Ct
26	S	St	NA	Ct	S	St	NA	Ct
27	St	St	NA	Ct	S	St	NA	Ct
28	S	St	NA	Ct	Ct	Ct	MA	Ct
29	S	St	NA	Ct	St	St	NA	Ct
30	S	St	NA	Ct	Ct	Ct	MA	Ct
31	S	St	NA	Ct	Ct	Ct	MA	Ct
32	Ct	St	NA	Ct	Ct	Ct	MA	Ct
33	Ct	St	NA	Ct	Ct	Ct	MA	Ct
34	S	St	NA	Ct	Ct	St	NA	Ct
35	St	St	NA	Ct	Ct	Ct	MA	Ct

S^*^: Saturated Ct^**^: Corrosion tendency St^***^: Scaling tendency NA^*^: Non-Aggressive MA^**^: Moderately Aggressive.

## 2 Experimental design, materials and methods

### 2.1 Description of study area

Divandarreh, one of the cities of Kurdistan province, located at west of Iran (35°54′N, 47°01′E). The city area is about 4203 km^2^ (approximately 15% of Kurdistan province area). The highest and lowest temperatures of air are 32 °C and −20 °C, respectively. According to the latest population census in Iran (2016), Divandarreh population was 81,000 persons.

### 2.2 Determine of water characteristics

To assessment of physiochemical parameters of the drinking water in rural area of Divandarreh province, 35 villages selected as sampling points. The samples were collected in rainy and dried seasons in 2015. The collected samples were delivered to laboratory in suitable conditions. After that, the samples were analyzed according to the standard methods for water and wastewater [1], [2], [3], [4], [5], [6], [7]. Measurement of temporary hardness, calcium, magnesium and chloride were carried out using titration method. Concentrations of fluoride, nitrate, and sulfate were determined by DR5000 spectrophotometer [1]. The EC and pH were analyzed with (Jenway 470 Conductivity meter) and pH meter (model Jenway 350), respectively.
